# Ternary zinc–tin-oxide nanoparticles modified by magnesium ions as a visible-light-active photocatalyst with highly strong antibacterial activity†

**DOI:** 10.1039/d4na00811a

**Published:** 2024-11-11

**Authors:** Alaa Kamo, Ozlem Ates Sonmezoglu, Savas Sonmezoglu

**Affiliations:** a Nanotechnology R&D Laboratory, Karamanoglu Mehmetbey University 70100 Karaman Turkey svssonmezoglu@kmu.edu.tr; b Department of Bioengineering, Karamanoglu Mehmetbey University 70100 Karaman Turkey; c Department of Metallurgical and Materials Engineering, Karamanoglu Mehmetbey University 70100 Karaman Turkey

## Abstract

Hospital-acquired infections (HAIs), especially nosocomial infections caused by antibiotic-resistant bacteria, are one of the most pressing health problems in all societies. Therefore, there is an urgent need to develop novel disinfection methods as alternatives to antibiotics to act against multidrug-resistant bacterial strains. Even though the photocatalytic disinfection phenomenon has been considered as a viable alternative compared to other proposed solutions, there is still a need to develop innovative functional materials for improving its efficacy under visible light to have a comparable impact to UV radiation. To boost the antibacterial efficacy under visible light, herein, we developed hydrothermally ternary zinc–tin-oxide (Zn_2_SnO_4_) nanoparticles modified with magnesium (Mg^2+^) ions at different doping ratios (0.5%, 1.0%, 1.5%, and 2.0%) as a photocatalytic disinfection agent and utilized it for the first time to kill Gram-negative (*E. coli*) and Gram-positive (*S. aureus*) pathogens that cause nosocomial infections. Moreover, we also explored how these materials interact with organic pollutants in the presence of visible light. Mg^2+^ cationic ions significantly enhanced the photocatalytic efficiency of ZTO nanoparticles under visible light to achieve 98% degradation of RhB dye in just 100 min, and rapidly produced numerous hydroxyl radicals as the main reactive oxygen species (ROS) responsible for the degradation, playing a key role in the nanoparticles impressive disinfection efficacy against these pathogenic bacteria. More importantly, Mg_1.5_@ZTO nanoparticles could effectively kill 99.76% of *E. coli* and 96.96% of *S. aureus* within only 1 h under visible light due to their smaller particle size, larger surface area, low recombination rate and greater ROS generation with oxygen vacancies. This research suggests that Mg-doped ZTO nanoparticles might be a viable and highly effective photocatalytic antibacterial agent candidate for future commercialization in healthcare and environmental applications.

## Abstract 2

This research not only provides a novel strategy for developing ternary magnesium-doped zinc–tin-oxide photocatalysts, but also unveils their potential in healthcare and environmental applications for future commercialization.

## Graphical abstract

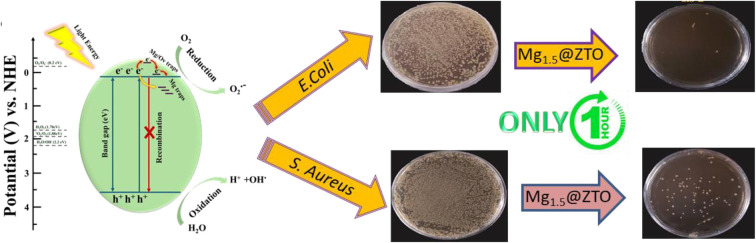

## 1. Introduction

Hospital-acquired infections (HAIs), also known as nosocomial infections, have become a global public health concern. The widespread and uncontrolled use of antibiotics, believed to be an effective method for treating these infections, is a serious concern as it causes drug resistance in bacteria. Therefore, there is an urgent need to develop novel antibacterial agents. With the advancement of nanotechnology, nanoparticles are often used as an alternative to conventional antibiotics. Nanoparticles show antibacterial activity through three different mechanisms: (i) physical interaction, (ii) ion release, and (iii) the production of reactive oxygen species (ROS).^1,2^ Nanoparticles penetrate the cell by adhering to the cell membrane through physical interaction, causing a leakage of cell inclusions and the degradation of vital components for the bacterium (such as protein, DNA, and enzymes).^3^ During ion release, metal ions interact directly with the mercapto (SH), amino (NH), and carboxylic (COOH) functional groups of macromolecules (proteins and nucleic acids) to inhibit enzymatic activity, alter the cellular structure, and interfere with normal physiological processes.^4^ Another antibacterial mechanism of nanoparticles is through the production of ROS and their photocatalytic activity. ROS cause cellular damage by oxidizing polyunsaturated fatty acids and amino acids. The free radicals produced during the photocatalytic process can cause lipid peroxidation, which ruptures the cell membrane and inhibits the growth of bacteria.^5–7^ ROS can also attack proteins and interfere with the activity of certain periplasm enzymes needed to maintain the life processes of bacterial cells.^8^

Nanoparticles exhibiting photocatalytic antibacterial properties can be classified as metal framework-based materials,^9,10^ noble metals,^11,12^ carbon-based materials,^13^ metal oxides,^14–16^ and chalcogenides.^17,18^ Among these, metal oxides are noted as up-and-coming candidates due to their non-toxic nature and high thermal stability.^19^ Binary metal oxides, such as TiO_2_, ZnO, and SnO_2_, are considered the most promising photocatalytic antibacterial agents. However, they require UV light, which is harmful to human health, for exhibiting photocatalytic antibacterial activity due to their large band gap. As an alternative to binary metal oxides, ternary oxide nanoparticles show better stability in aqueous conditions,^20,21^ which is why research on them is growing daily. Among the ternary oxide materials, zinc–tin oxide (Zn_2_SnO_4_, or ZTO), also known as zinc stannate, is an n-type semiconductor with an A_2_BO_4_ cubic inverse spinel crystalline structure,^22,23^ and it is known for its wide band gap (3.6 eV), good stability under adverse conditions, and high mobility already achieved for specific nanostructures (112 cm^2^ V^−1^ s^−1^).^24^ The above-mentioned properties have increased the use of ZTO as a photocatalytic antibacterial agent. Although ZTO nanoparticles exhibit high photocatalytic antibacterial activity under UV irradiation due to their wide band gap, their low absorption coefficient in the visible region and high recombination rate of electron–hole pairs limit their photocatalytic antibacterial activity under visible light. It is obvious that the doping approach is undoubtedly one of the most effective ways to overcome these limitations. Therefore, we focused on modifying ZTO nanoparticles with magnesium (Mg) elements as cationic dopants and exploring how they affect their photocatalytic and antibacterial properties. We chose magnesium as a cation dopant for the following reasons: (i) Zn (74 pm) and Mg (72 pm) have comparatively close ionic radii and both have 2+ valence states, therefore substituting Mg in to the ZTO crystal lattice in place of Zn will not cause a disorder in the crystal structure, (ii) the difference in electronegativity, which is another crucial factor in dopant preferences, between Mg (1.31) and Zn (1.65) is rather small, implying that the band gap of ZTO nanoparticles may shrink with Mg doping, (iii) even though it has the largest absorption ability around 460 nm, the fact that the Mg element displays a strong absorption ability up to about 600–700 nm when bound to the oxygen atom, meaning that the incorporation of Mg will improve the visible-light sensitivity of ZTO,^25^ (iv) Mg^2+^ cationic ions will damage the bacterial membrane and make the bacteria more vulnerable, promoting an increase in antibacterial activity,^26–28^ and (v) Mg^2+^ ions will increase the permeability of bacterial cell membranes, resulting in cell inclusion leakage, meaning that Mg will play a more effective role in the rapid destruction of bacteria.^29^ Surprisingly and to the best of our knowledge, there is just one work in the literature that focuses on the photocatalytic activity of Mg-doped ZTO nanoparticles, and there is no research on their antibacterial abilities. In the one work in the literature, Mg-doped ZTO nanoparticles displayed satisfactory photocatalytic activity under visible light.^25^ However, excessive doping rates led to the photonic behavior-triggered formation of numerous defects in the crystal structure, making it impossible to observe the expected high performance. Therefore, we emphasize efforts to fully comprehend how ZTO nanoparticles with lower Mg doping rates would differ in terms of their photocatalytic activity as well as the underlying mechanism behind their antibacterial activity.

For the first time, we evaluated the impact of hydrothermally Mg-doped ZTO nanoparticles on *S. aureus* and *E. coli* pathogens causing nosocomial infections under visible light. Furthermore, the effects of these nanoparticles on the degradation of a representative organic pollutant, namely rhodamine B dye, were examined in detail using both UV and visible light. The degradation efficiency of ZTO nanoparticles under visible light was improved by more than two-fold through Mg doping. Furthermore, the Mg_1.5_@ZTO nanoparticles showed significant antibacterial activity against *E. coli* and *S. aureus* bacterial strains, with 99.76% and 96.96% inactivation efficiencies, respectively. These findings reveal that the Mg-doped ZTO nanoparticles have good biocompatibility and negligible toxicity based on the requirements of the human body for magnesium and zinc elements, further indicating their potential for application in healthcare as effective antibacterial agents.

## 2. Experimental section

### 2.1. Synthesis of the nanoparticles

Pure and Mg-doped ZTO nanoparticles were synthesized *via* a modified hydrothermal method.^30^ In a typical process, 0.008 mmol zinc acetate dihydrate and 0.004 mmol tin(iv) chloride pentahydrate solutions were prepared separately in 20 mL deionized water. The as-prepared solutions were then combined, and a bright white solution was obtained. Then the pH value was adjusted to 9 using NaOH solution. The solution was then autoclaved at 180 °C for 24 h. Following the hydrothermal process, the precipitate was collected and washed with deionized water. Afterward, the nanoparticles were dried in the oven for 6 h at 100 °C. The same process was used to synthesize Mg-doped ZTO nanoparticles using an appropriate amount of Mg(NO_3_)_2_·6H_2_O. ZTO nanoparticles doped with 0, 0.5%, 1.0%, 1.5%, and 2.0% magnesium were identified as pure ZTO, Mg_0.5_@ZTO, Mg_1.0_@ZTO, Mg_1.5_@ZTO, and Mg_2.0_@ZTO, respectively.

### 2.2. Characterizations of the nanoparticles

The crystal structures of the pure and Mg-doped ZTO nanoparticles were analyzed by X-ray powder diffraction (Bruker D8 Advanced). The reflectance and absorption spectra were recorded using a UV-vis spectrophotometer (Shimadzu UV-2600). The defects in the nanoparticles were analyzed by Raman spectroscopy (inVia, RENİSHWA). The chemical bonds were examined by FTIR analysis (Bruker VERTEX 70). Photoluminescence (PL) spectra were recorded using a fluorescence spectrophotometer (HITACHI Fluorescence Spectrophotometer F-7100). X-Ray photoelectron spectroscopy (Thermo Scientific K-Alpha) was used to study the surface electronic states and related atomic compositions. The surface morphology, particle size, and stoichiometry of the nanoparticles were studied by scanning electron microscopy (SEM, HITACHI SU5000). An FEI TALOS F200S TEM 200 kV instrument was used for the TEM analysis. The specific surface area and pore size of the pure and Mg-doped ZTO nanoparticles were examined by measuring the N_2_ adsorption–desorption isotherms with a Micromeritics/TriStar II Plus system.

### 2.3. Photocatalytic activity

The photocatalytic activities of the pure and Mg-doped ZTO were determined under UV and visible light irradiation. For UV irradiation, 12 UV-C (96 W) lamps were used. The visible light source was an Osram Dulux lamp (23 W) with an ultraviolet-blocking filter (Thermolab FEL0400). First, the obtained nanoparticles as photocatalyst were dispersed in 100 mL of an aqueous suspension of RhB. The mixture was then stirred with a magnetic stirrer for 10 min in the dark to establish adsorption–desorption equilibrium. Then, 3 mL aliquots were periodically taken out and the absorbance was measured. For the reusability experiments, the Mg_1.5_@ZTO nanoparticles were washed several times with ddH_2_O and dried at 100 °C for three hours prior to each re-use.

### 2.4. Scavenger experiments

Scavenger tests were carried out in the presence of Mg_1.5_@ZTO nanoparticles according to the protocol given in ref. 21. In the scavenger tests, EDTA-Na_2_, 4-benzoquinone, *T*-butyl alcohol, and AgNO_3_ were used to capture holes (h^+^), superoxide radicals (O_2_^−^), hydroxyl radicals (˙OH), and electrons (e^−^), respectively. The experimental procedure used for this test was similar to that used for the photocatalytic activity study.

### 2.5. Antibacterial activity

The antibacterial activity of all nanoparticles was evaluated under visible light using by the colony count method. Two bacterial strains were used in the antibacterial tests: Gram-negative (*Escherichia coli*-ATCC 25922) and Gram-positive (*Staphylococcus aureus*-ATCC 25923). *E. coli* and *S. aureus* bacterial cells were cultured in LB medium and incubated for 18 h. Then, the bacterial suspensions were diluted until 10^−6^ CFU mL^−1^ with 0.85% NaCl solution. Afterward, 1000 μL pure and Mg-doped ZTO nanoparticles (1 mg mL^−1^) were added. The bacterial suspensions were then incubated for different times under visible light. After incubation, the bacteria were cultured in a solid LB medium and incubated at 37 °C for *E. coli* and 35 °C for *S. aureus*. At the end of this period, the bacteria were enumerated.

## 3. Results and discussions

**Fig. 1:**
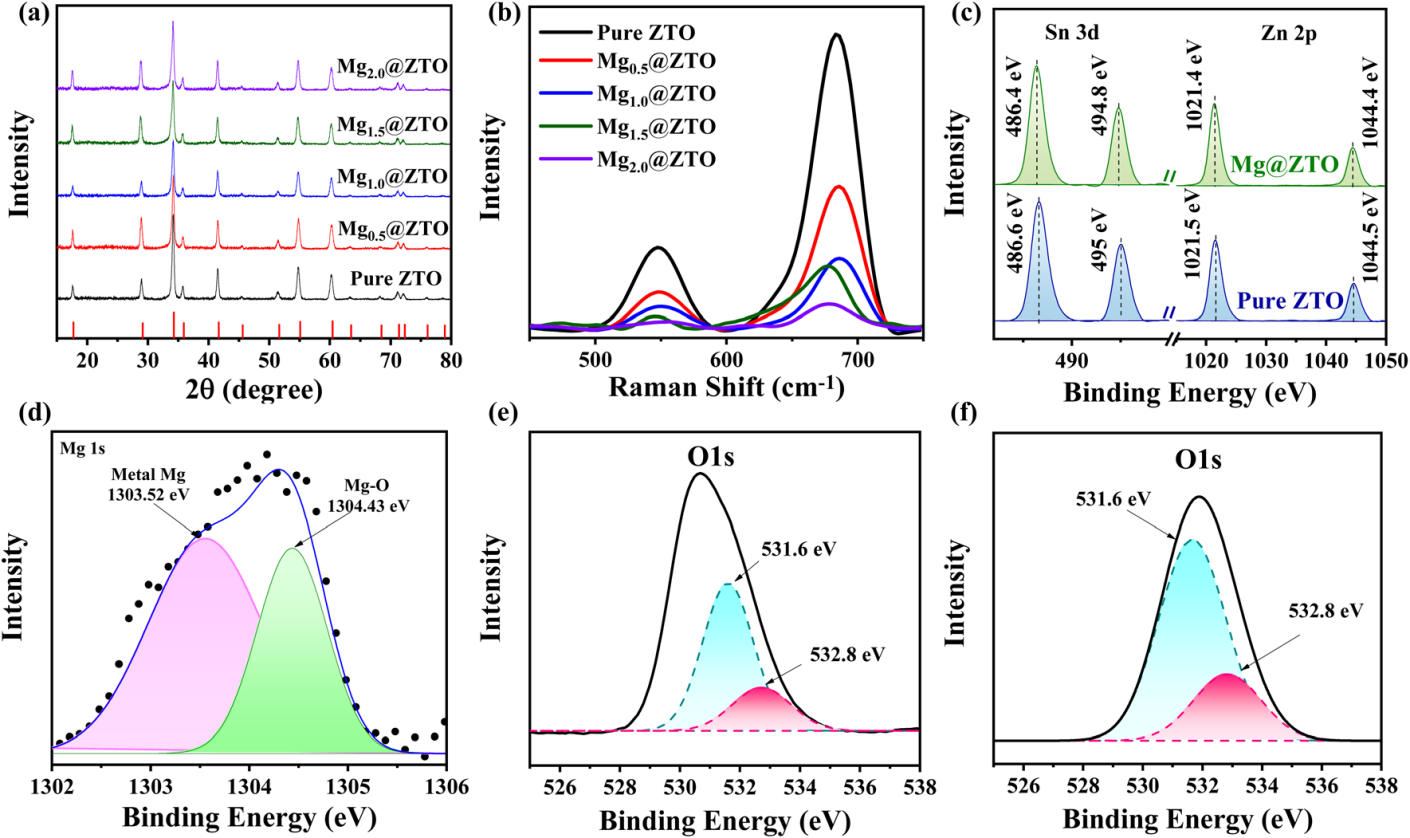
(a) XRD patterns, (b) Raman spectra, and (c) XPS Zn 2p and Sn 3d spectra of pure and Mg-doped ZTO nanoparticles, (d) Mg 1s spectrum of Mg-doped ZTO nanoparticles, (e) O 1s spectrum of pure ZTO, and (f) O 1s peak of Mg_1.5_@ZTO.

XRD spectroscopy was used to assess the crystal structures of the pure and Mg-doped ZTO nanoparticles (Fig. 1(a)). The diffraction peaks of the pure ZTO nanoparticles were located at 17.68°, 28.91°, 34.19°, 35.76°, 41.47°, 45.60°, 51.48°, 54.81°, 60.26°, 63.42°, 68.33°, 71.22°, 72.11°, 75.98°, and 78.52° corresponding to the (111), (220), (311), (222), (400), (331), (422), (511), (440), (531), (620), (953), (862), (344), and (271) planes of the inverse spinel structure (PDF#00-024-1470), respectively. Fig. 1(a) illustrates that incorporating less than 2% Mg dopant resulted in a negligible decrease in peak intensities and no secondary phase formation, suggesting that strain-induced disorder did not exist in the crystal structure.^31^ Conversely, incorporating 2% Mg dopants caused the peak intensity to significantly decrease, indicating that the crystal structure was beginning to deteriorate. To verify this finding, the lattice parameters of the pure and Mg-doped ZTO nanoparticles were calculated according to Bragg's law.

1 2*d* sin *θ = nλ*

Here, *d* is the distance between the planes, *θ* is the Bragg angle, *n* = 1 for the first order diffraction order, and *λ* is the wavelength of the X-ray source (1.5406 Å). The lattice parameters of the pure ZTO, Mg_0.5_@ZTO, Mg_1.0_@ZTO, Mg_1.5_@ZTO, and Mg_2.0_@ZTO nanoparticles were calculated as 8.64, 8.579, 8.576, 8.553, and 8.571 Å, respectively. The lattice parameters of ZTO decreased with Mg doping, indicating that the Mg^2+^ ions were localized in the crystal lattice.

The chemical bonding and functional groups of the pure ZTO and Mg-doped ZTO nanoparticles were investigated in detail by FTIR characterization. As shown in Fig. S1,† four peaks were observed at 390, 505, 1018, and 3416 cm^−1^, corresponding to Zn–O, Sn–O, Sn–O–Zn, and OH bonds.^32,33^ The same peaks were present in all Mg-doped ZTO nanoparticles, indicating that the addition of Mg as a dopant did not lead to any change in the chemical bonding structure of ZTO.

Raman spectroscopy was employed to examine the molecular vibrational modes in both the pure and Mg-doped ZTO nanoparticles. Fig. 1(b) shows the Raman spectra of both nanoparticles. Two well-defined Raman groups could be observed at 545 and 684 cm^−1^, corresponding to F_2g_ and A_1g_ symmetries (Fig. 1(b)). Furthermore, no peaks for Mg^2+^ ions were found in the Raman spectra, as confirmed by the XRD analysis. The peak above 600 cm^−1^ represented the stretching vibrations of the metal–O (Zn/Sn–O) bonds in tetrahedral MeO_4_.^34^ The F_2g_ mode (526–556 cm^−1^) is exclusive to the inverse spinel structure and corresponds to the vibrations of Zn and Sn cations in the octahedral regions.^35,36^

The surface chemical compositions and oxidation states of the fundamental elements of the pure and Mg_1.5_@ZTO nanoparticles were analyzed by XPS. Fig. S2† presents survey scans of both nanoparticles, indicating the presence of Zn, Sn, O, and Mg elements. The high-resolution patterns of Zn 2p and Sn 3d in both the ZTO nanoparticles are shown in Fig. 1(c). The binding energies of Zn 2p_3/2_ and Zn 2p_1/2_ in Mg-doped ZTO were 1021.4 and 1044.4 eV, respectively, representing a 0.1 eV shift from the binding energies (1021.5 and 1044.5 eV) in pure ZTO. The binding energies of Sn 3d_5/2_ and Sn 3d_3/2_ in both nanoparticles were at 486.4 and 494.8 eV for Sn^4+^, indicating that there was no shift compared to the pure ones. This clearly reveals that Mg was replaced by the Zn atom in the crystal lattice and not by the Sn atom. The underlying reason for this shifting is because Mg cations have lower electronegativity than Zn cations, which strengthens the electronic density on the surface of the Zn atom, lowering the binding energy of Zn 2p. The Mg 1s signal in Fig. 1(d) confirms that the Mg cations were effectively incorporated into the ZTO structure. The Mg 1s peak consisted of two principal components, one at 1304.43 eV related to the Mg–O bond and one at 303.52 eV representing metallic magnesium.^37^ The high-resolution patterns of O 1s in the pure and Mg-doped ZTO nanoparticles are shown in Fig. 1(e and f). Meanwhile, the O 1s spectrum was separated into two peaks: a peak at 531.6 eV, corresponding to oxygen-related vacancies in the crystal lattice, and a peak at 532.8 eV, corresponding to weak bonding hydrated species –OH on the surface of ZTO. The incorporation of Mg cations caused a considerable improvement in the number of oxygen vacancies in the ZTO structure. The presence of oxygen vacancies leads to the formation of new donor levels as trap centers between the valence and conduction bands, which slows the recombination rate and prevents the nonradiative recombination, thus improving the photocatalytic activity.^38^ Furthermore, this scenario encourages the production of reactive oxygen species (ROS), which enhances the excellent photocatalytic activity of ZTOs under both UV and visible light.^39,40^

**Fig. 2:**
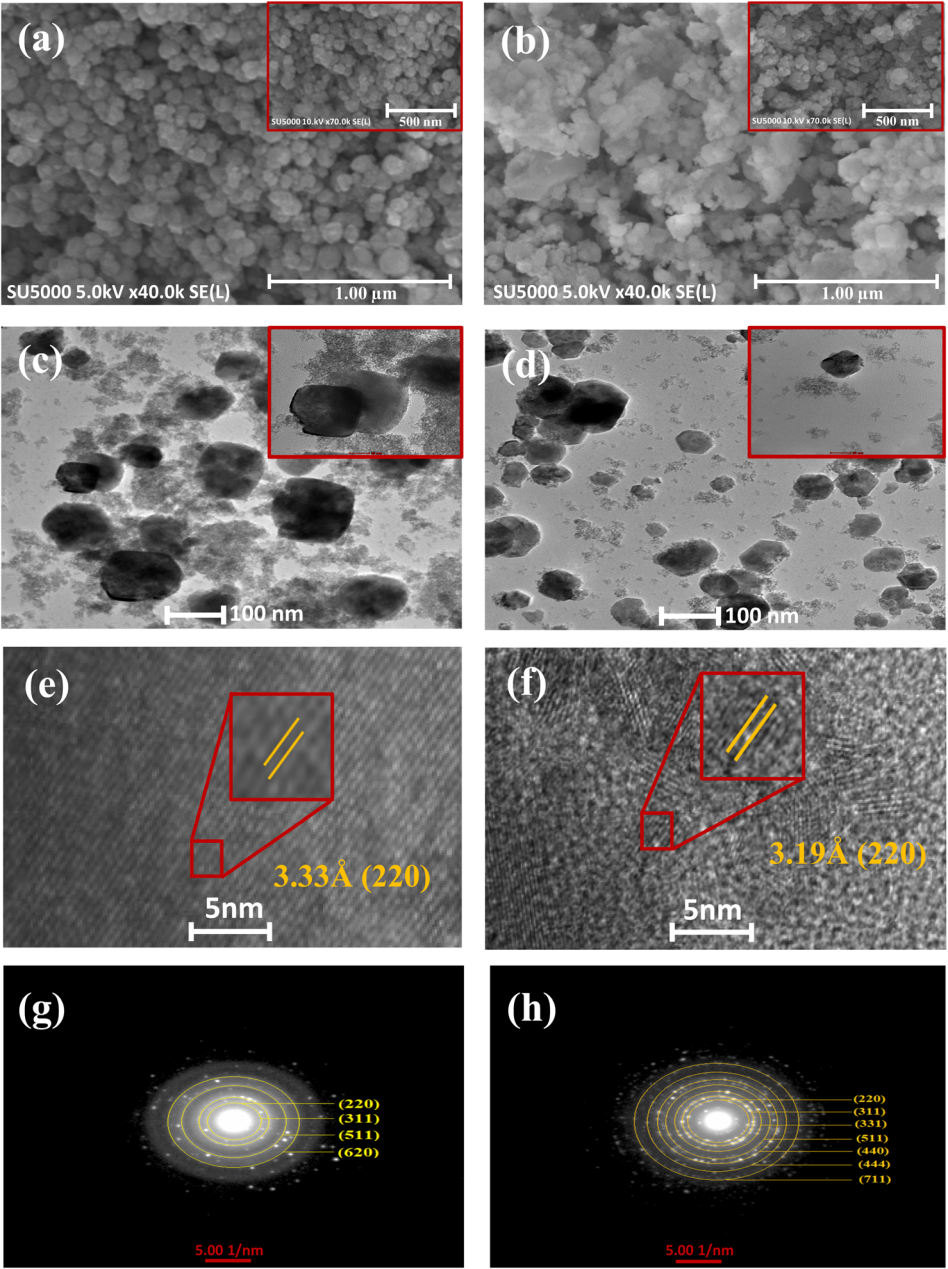
FESEM images of (a) pure and (b) Mg_1.5_@ZTO, TEM images of (c) pure and (d) Mg_1.5_@ZTO, HR-TEM of (e) pure and (f) Mg_1.5_@ZTO, SAED analysis of (g) pure and (h) Mg_1.5_@ZTO.

Typical field emission scanning electron microscopy (FESEM) images of the pure and Mg-doped ZTO are presented in Fig. 2(a, b) and S3(a–c).† Regardless of the doping process with magnesium or not, both nanoparticles exhibited a spherical-like morphology. The presence of magnesium in the nanoparticles was verified by elemental chemical analysis using energy-dispersive X-ray spectroscopy (EDS) and mapping methods, and the results are shown in Fig. S4 and S5.† It was found that the pure ZTO nanoparticles had an atomic ratio of 2 : 1 : 4. Similar stoichiometric rates were applicable for the varied magnesium concentrations. Furthermore, elemental mapping analysis based on energy-dispersive X-ray spectroscopy (EDS) in Fig. S5† revealed a rather uniform distribution of Zn, Sn, O, and Mg elements across the nanoparticles. Notably, transmission electron microscopy (TEM) measurements of the pure and Mg-doped ZTO nanoparticles from Fig. 2(c, d) and S3(d–f)† revealed a similar morphology as evidenced in the FESEM images; however, the size of the nanoparticles in ZTO was reduced dramatically from approximately 77 nm to 42 nm after Mg incorporation. This phenomenon may be explained by the fact that the dopant elements act as nucleation sites during the synthesis of nanoparticles, and the number of nucleation sites increases as the energy required for the particles to form decreases, leading to the formation of more small particles. Based on the HRTEM images from Fig. 2(c, d) and S6(a–c)†, one can see that pure and Mg-doped ZTO nanoparticles have well defined lattice fringes with interplanar spacings of 0.333 and 0.319 nm, which correspond to the (200) crystal planes, respectively. Furthermore, pure ZTO exhibited perfect atomic arrangements inside the crystal lattice, but it also had rich grain boundaries depending on the presence of different dislocation lines after Mg inclusion. This might result from heterogeneous nucleation-induced distortions in the crystal structure or from aggregation of the nanocrystals. As a result, the creation of larger grain boundaries enhances the specific surface area and the accessibility of the active sites for photocatalytic processes.^41^ The circular rings in the selected-area electron diffraction (SAED) pattern for both nanoparticles (Fig. 2(g, h) and S6(d–f)†) confirmed their polycrystalline nature.

**Fig. 3:**
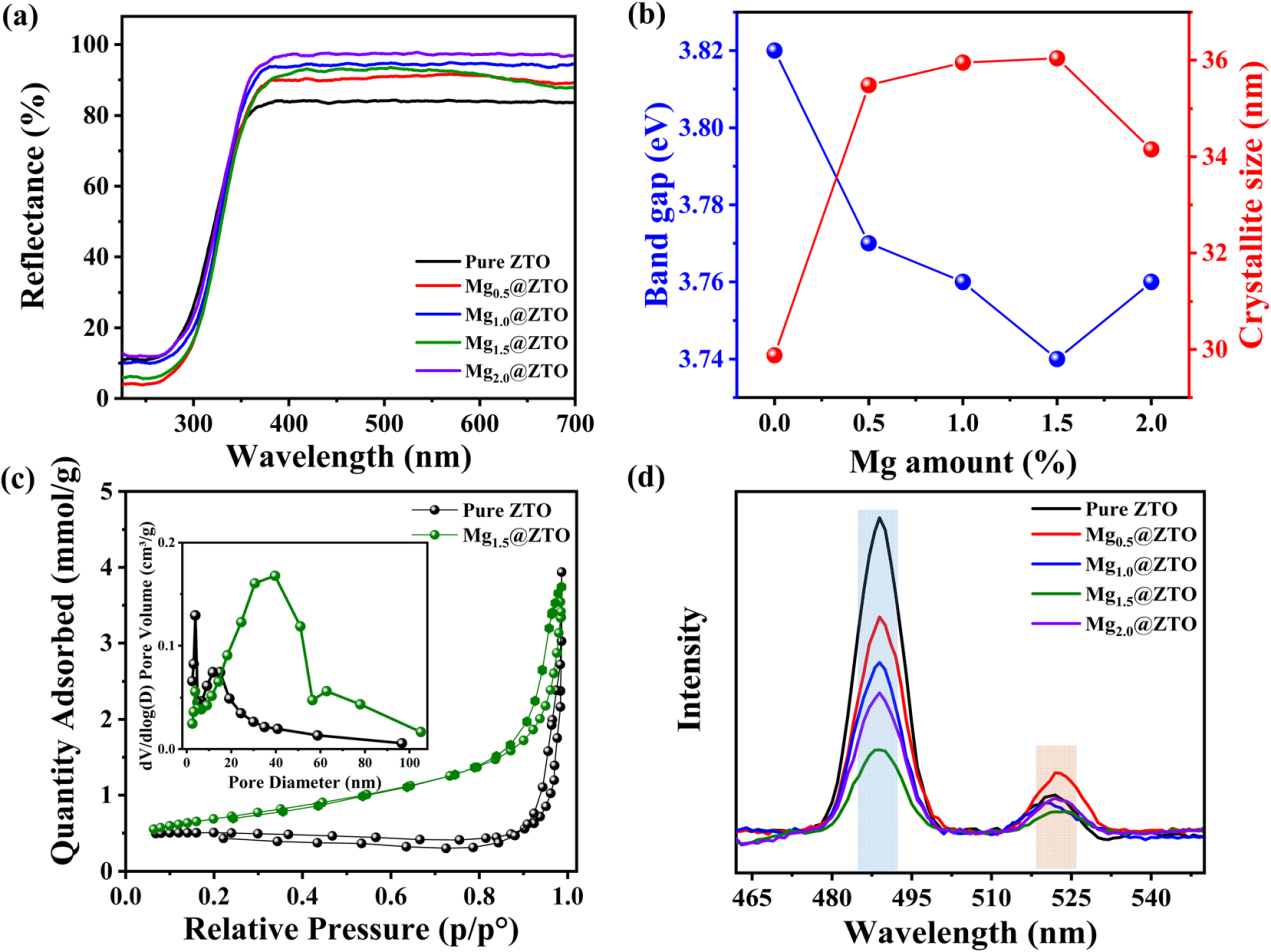
(a) DRS spectra and (b) band gap and crystallite size of the pure and Mg-doped nanoparticles. (c) N_2_ adsorption–desorption isotherm curve and pore diameter of the pure and Mg_1.5_@ZTO. (d) PL spectra of all the nanoparticles.

To evaluate the optical properties of the nanoparticles, as shown in Fig. 3(a), we examined the samples' diffuse reflectance spectra ranging from 250 to 700 nm. Based on Kubelka–Munk theory (Fig. S7†), the band gaps of the pure ZTO, Mg_0.5_@ZTO, Mg_1.0_@ZTO, Mg_1.5_@ZTO, and Mg_2.0_@ZTO nanoparticles were calculated to be 3.82, 3.77, 3.76, 3.74, and 3.76 eV, respectively, and are displayed in comparison with their crystallite size in Fig. 3(b). The reason for this decrease in the band gap with Mg incorporation could be explained by the Moss–Burstein effect.^42,43^Fig. 3(c) and S8(a–c)† show the N_2_ adsorption–desorption isotherms of the pure and Mg-doped ZTO nanoparticles. The surface areas of the pure ZTO, Mg_0.5_@ZTO, Mg_1.0_@ZTO, Mg_1.5_@ZTO, and Mg_2.0_@ZTO nanoparticles were calculated as 37.7, 38.7, 39.6, 55.1, and 38.2 m^2^ g^−1^, respectively. Both nanoparticles types exhibited type III isotherms,^44^ and Mg doping appeared to enhance the surface area of the ZTO nanoparticles, leading to more reaction-active sites and a considerable improvement in photocatalytic efficacy by raising the reaction rate.^45^ The pore width of the photocatalyst is another factor that influences its photocatalytic and antibacterial properties. The pore diameters of pure ZTO, Mg_0.5_@ZTO, Mg_1.0_@ZTO, Mg_1.5_@ZTO, and Mg_2.0_@ZTO were 65.4, 30.1, 38.08, 39.7, and 56.3 nm, respectively, as shown in Fig. 3(c) and S8(d–f).† The increase in pore widths after Mg inclusion into the host matrix will have a favorable effect on their photocatalytic and antibacterial activities by enhancing the separation efficiency of photogenerated charge carriers.^46,47^ Photoluminescence spectroscopy (PL) was utilized to elucidate the separation and recombination of photogenerated electrons and holes. In Fig. 3(d), two peaks could be observed in the PL spectrum at 488 and 522 nm associated with oxygen vacancies and Zn/Sn stoichiometry, respectively.^48^ The PL intensity of the Mg_1.5_@ZTO nanoparticles was much lower than that of the others, indicating that the insertion of optimal Mg ions may increase the separation efficiency of photo-induced carriers through shallow centers on the surface while simultaneously suppressing nonradiative recombination. Moreover, a few works in the literature have claimed that the decline in PL density may be due to the presence of more oxygen vacancies within the crystal lattice,^49,50^ confirming that the insertion of magnesium ions facilitates the formation of more active sites, which encourages the enhancement of the photocatalytic and antibacterial properties.

**Fig. 4:**
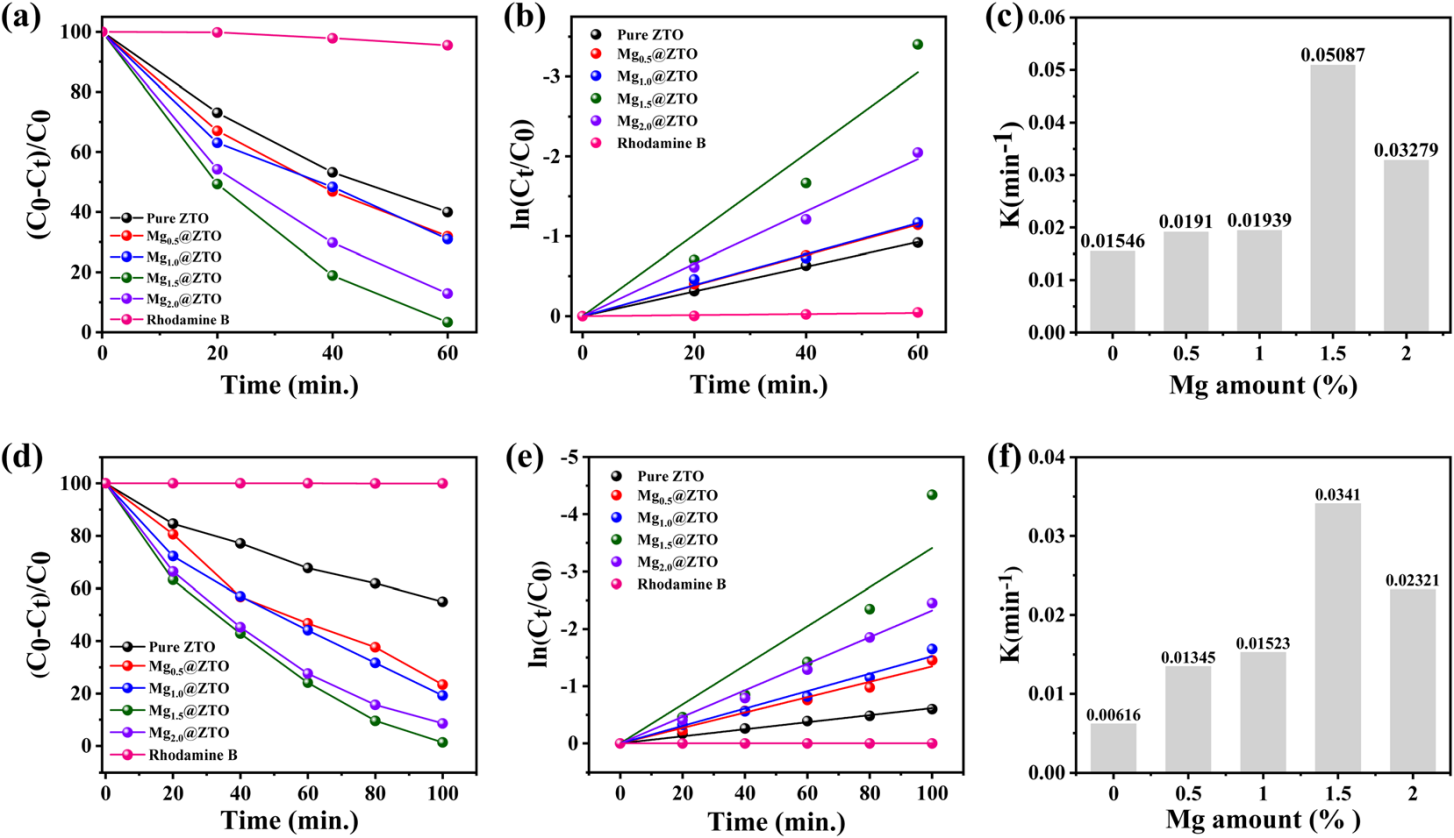
(a) Degradation rates, (b) kinetic parameters, and (c) rate constant of pure and Mg-doped ZTO nanoparticles under UV-light irradiation. (d) Degradation rate, (e) kinetic parameters, and (f) rate constant of pure and Mg-doped ZTO nanoparticles under visible-light irradiation.

The photocatalytic activities of the pure and Mg-doped ZTO nanoparticles were evaluated under UV and visible-light illumination (*λ* ≥ 400 nm) and the results are displayed in Fig. 4. To establish the depth of light penetration necessary for the reaction,^51,52^ we first began with experiments to identify the optimal photocatalyst dose. To optimize the amount of photocatalyst, we experimented with four different dosages (60, 90, 120, and 150 mg) of ZTO nanoparticles and found that the highest photocatalytic activity was achieved with 90 mg of ZTO catalyst, as shown in Fig. S9.† Unless otherwise specified, this quantity of photocatalyst was utilized in all the further photocatalytic experiments. Herein, we employed rhodamine (RhB) dye as an organic pollutant to assess the photocatalytic activity. Initially, we explored how Mg cationic ions affect the ability of ZTO nanoparticles to degrade organic pollutants when exposed to UV-light irradiation (Fig. S10†). As can be seen in Fig. 4(a), pure ZTO degraded only about 60% of RhB dye in 60 min under UV-light irradiation, whereas the Mg_1.5_@ZTO nanoparticles degraded more than 96% in the same period. The rate constant (*K*) of the photocatalytic process was determined using pseudo-first-order kinetics, and Fig. 4(b) shows a perfect linear correlation between the logarithmic concentration and time. Furthermore, as shown in Fig. 4(c), the reaction rate constant for the Mg_1.5_@ZTO nanoparticles reached 0.05087 min^−1^, which was 3.8 times greater than that of pure ZTO (0.01546 min^−1^). The photocatalytic activities of the pure and Mg-doped ZTO nanoparticles were also evaluated under visible-light irradiation, and the varying of the degradation efficiencies with time are given in Fig. S11.† More importantly, as shown in Fig. 4(d), Mg-ZTO could degrade over 98% of RhB dye in just 100 min, whereas pure ZTO could degrade just 45% in the same time period. Fig. 4(e and f) show the kinetic parameters and rate constant for RhB degradation in the presence of the pure and Mg-doped ZTO nanoparticles under visible light. The reaction rate constant of the Mg_1.5_@ZTO nanoparticles was 0.0341 min^−1^, which was 5.5 times higher than that of pure ZTO. The Mg_1.5_@ZTO nanoparticles demonstrated remarkable photocatalytic activity under visible light, compared to the pure nanoparticles and other ones, owing to their low recombination rate, smaller size, and larger surface area, as evidenced by the above-mentioned experimental findings. The Mg_1.5_@ZTO nanoparticles exhibited impressive photocatalytic efficiency compared to similar works in the literature, as can be seen in Table S1,† which presents a summary of the literature on the photocatalytic and antibacterial activities of various cation- and anion-doped ZTO nanoparticles.

**Fig. 5:**
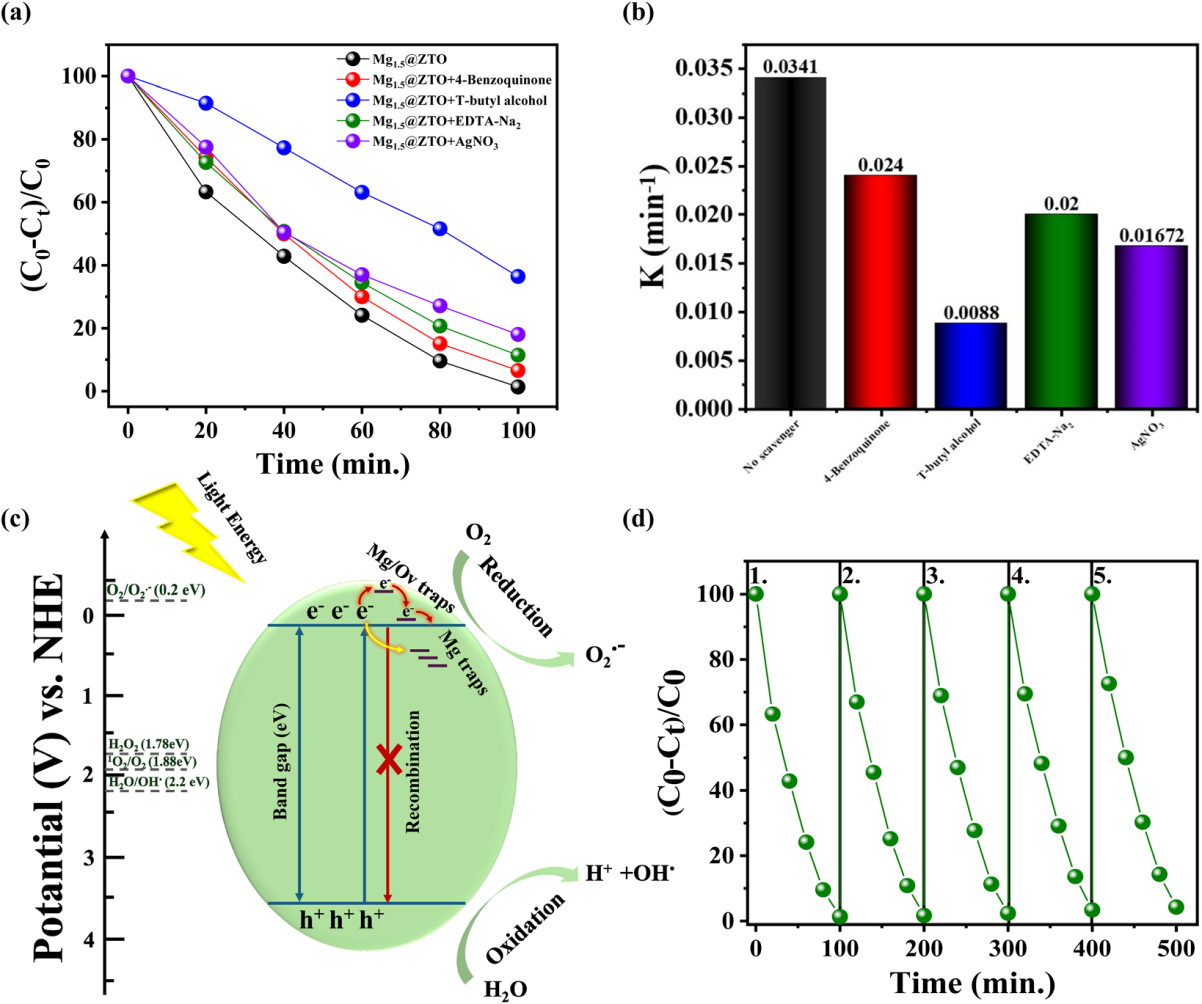
(a) Photocatalytic degradation rate of RhB and (b) photocatalytic rate constant using Mg_1.5_@ZTO nanoparticles in the presence of different free radical scavengers. (c) Possible photocatalytic mechanism of Mg_1.5_@ZTO nanoparticles and (d) the recycling ability of Mg_1.5_@ZTO nanoparticles for RhB degradation under visible-light irradiation.

A series of experiments using certain scavengers were conducted to understand the exact roles of the reactive oxygen species responsible for photocatalytic bacterial inactivation. Scavengers such as EDTA-Na_2_, 4-benzoquinone, *T*-butyl alcohol, and AgNO_3_ were typically employed using Mg_1.5_@ZTO nanoparticles under visible light to remove photogenerated h^+^, ˙O_2_^−^, ˙OH, and e^−^. The degradation efficiency of RhB dye reached 98.67% in the absence of scavengers, as shown in Fig. 5(a). As expected, the degradation efficiency was dramatically reduced in the presence of EDTA-Na_2_, 4-benzoquinone, *T*-butyl alcohol, and AgNO_3_ with values of 81.97%, 63.53%, 93.45%, and 88.57%, respectively. While the highest quenching impact occurred with the addition of *T*-butyl alcohol as shown in Fig. 5(b), the minimal change was noticed with AgNO_3_, implying that photogenerated holes had less impact among the primary reactive species in the photocatalytic and antibacterial process. It appears that ˙OH radicals played an active role in the photocatalytic reaction of Mg-doped ZTO nanoparticles compared to other ROS. This outcome can be interpreted as follows: (i) the release of hydroxyl radicals into the aqueous environment as a result of the reduction of H_2_O_2_, with free electrons attracted to the surface by oxygen vacancies, means that the H_2_O_2_ radicals are increased by the two ˙OH radicals occurring from the oxidation of photogenerated holes in the valence band due to the presence of more e^−^/h^+^ pairs with magnesium doping.^53,54^ (ii) The release of surface hydroxyl groups towards the bulk solution due to their easy desorption from the surface leads to their formation by the reaction of atomic oxygen species (*O), moving from the defect sites inside the bulk to the surface, with water molecules.^55^ Surprisingly, photogenerated electron and superoxide radicals appeared to be active in photocatalytic and antibacterial processes, even though ZTO is known to produce insufficient amounts of O_2_ due to its conduction band levels being below the O_2_/˙O_2_^−^ (0.2 eV) limit.^56^ This result shows that magnesium doping could create new energy levels through defects, such as Mg interstitial or oxygen vacancies on the surface near the conduction band, resulting in the upward movement of the conduction band. The photocatalytic mechanism of Mg_1.5_@ZTO nanoparticles can be explained by the following reactions: briefly, electron–hole pairs are generated when photons with enough energy are absorbed by the Mg_1.5_@ZTO catalyst. These pairs occur *via* a sequence of reductive or oxidative redox pathways in Fig. 5(c), including: (i) the photogenerated holes in the valence band reacting with water to form hydroxyl radicals, (ii) the photogenerated electrons in the conduction band reacting with oxygen to form superoxide radicals (˙O_2_^−^), which are subsequently protonated and a superoxide radical (˙HO_2_) is formed by the reaction between this radical and H^+^ originating from the breakdown of water, (iii) H_2_O_2_ is formed by the interaction of protonated superoxide radicals (˙HO_2_) and photogenerated holes, (iv) the formation of two hydroxyl radicals by the reaction of H_2_O_2_ with photogenerated electrons. More crucial than the well-known reactions is that we describe for the first time, direct observation of how defect-induced *O species and water react on a catalyst surface, providing an increase in ˙OH radicals, thereby revealing a chemical pathway for the formation of ˙OH radicals that was previously unreported for similar nanoparticles. As shown in Fig. 5(d), the degradation rates after five cycles were 98.96%, 98.33%, 97.65%, 96.61%, and 95.74%, indicating that Mg_1.5_@ZTO nanoparticles were very stable and repeatable performances.

**Fig. 6:**
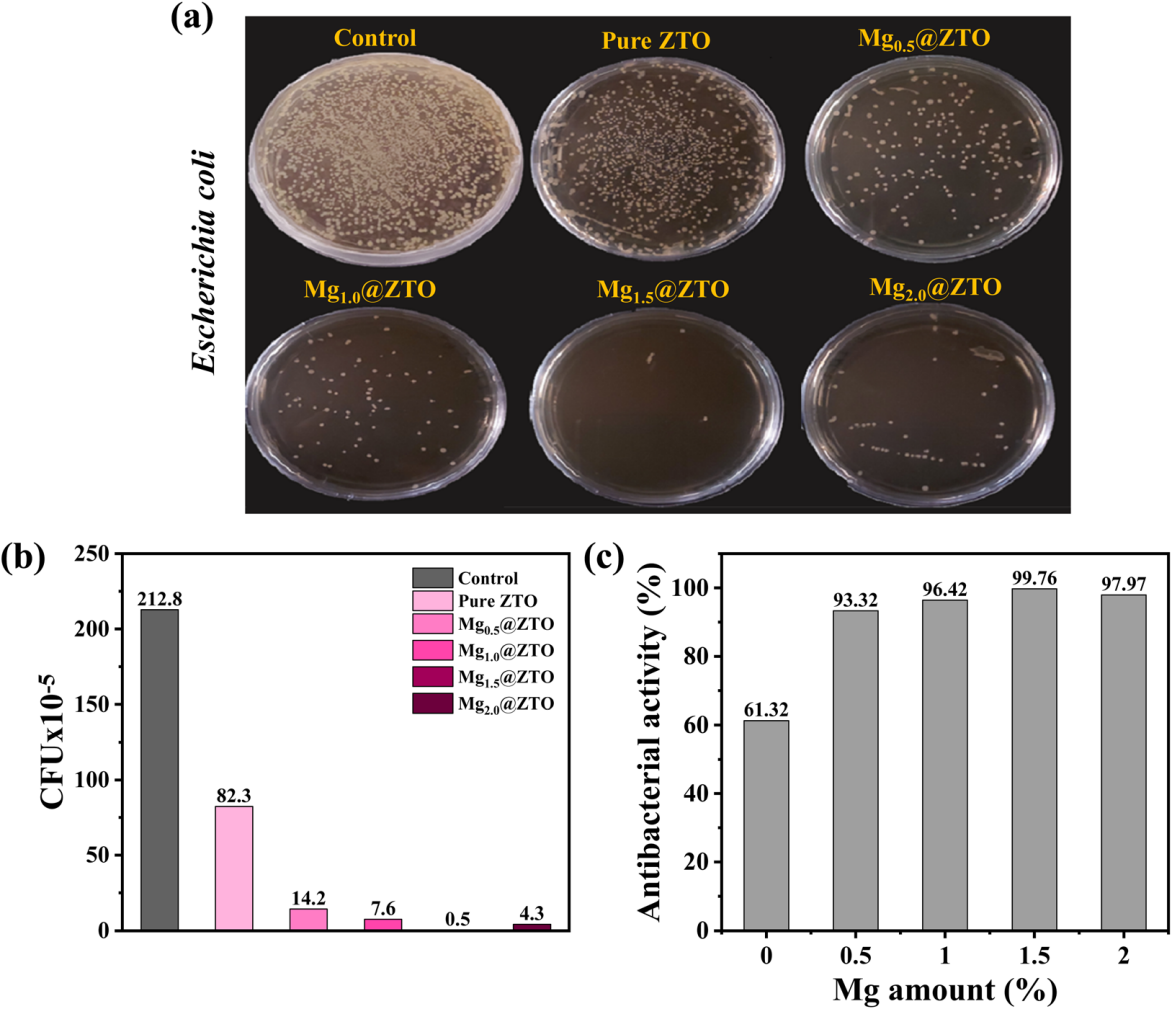
(a) Plate counting photographs, (b) cell viability of *E. coli* in the presence of pure and Mg-doped ZTO nanoparticles, and (c) antibacterial activity of pure and Mg-doped ZTO nanoparticles.

Prior to comprehensively assessing the antibacterial efficacy of the ZTO nanoparticles, the optimal quantity was first adjusted by changing the dosage of catalyst. As seen in Fig. S12,† the photocatalytic antibacterial activity was improved with increasing the catalyst dosage, equivalent to dosages of 500, 1000, and 2000 μL, respectively, and yielded the highest dose when using 1000 μL of catalyst. Therefore, 1000 μL of catalyst was the optimal dosage to prepare all the nanoparticles. Thereafter, we examined the antibacterial performance of the pure and Mg-doped ZTO nanoparticles under visible light for 1 h, and the results are shown in Fig. 6(a). It could be clearly seen from Fig. 6(a) that the survival rates of *E. coli* in the control group, not exposed to ternary-based oxide nanoparticles, were not significantly affected by 1 h exposure under visible irradiation; in contrast, the ZTO nanoparticles modified with 1% or more magnesium cationic dopant demonstrated impressive inactivation efficiency against *E. coli* bacteria under the same conditions. The reason why ZTO exhibited a low inactivation efficiency to *E. coli* at high magnesium dopants rates (2%) could be explained by the presence of larger particles along with the aggregation of ZTO. To quantitatively evaluate the antibacterial activities of the all nanoparticles against the *E. coli* bacteria strain, the colony-forming units and antibacterial activity percentages were examined in detail, as shown in Fig. 6(b and c). Based on these findings, Mg_1.5_@ZTO nanoparticles had the best inactivation efficiency of 99.76% against *E. coli* pathogens.

**Fig. 7:**
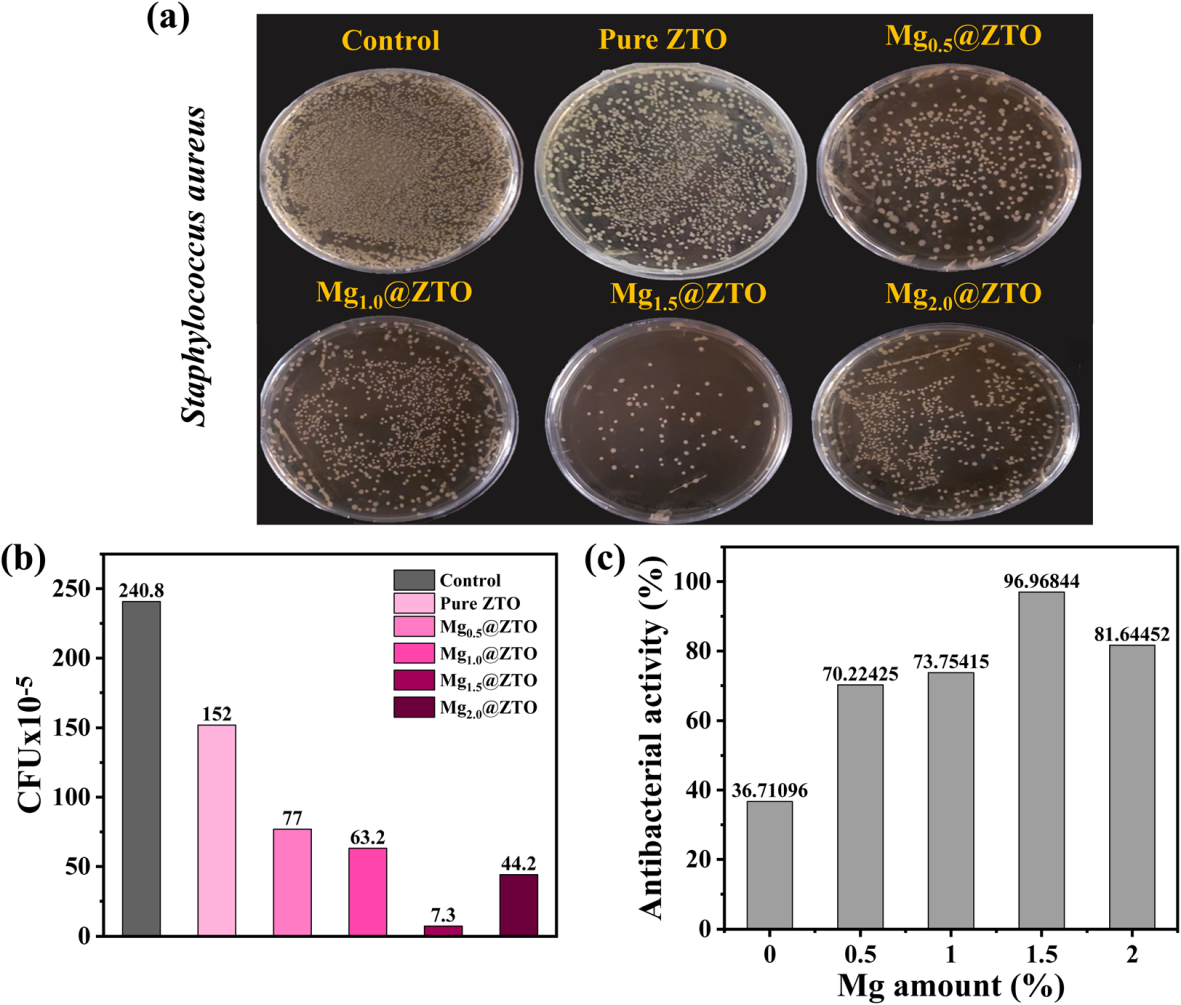
(a) Plate counting photographs, (b) cell viability of *S. aureus* in the presence of pure and Mg-doped ZTO nanoparticles, and (c) antibacterial activity of pure and Mg-doped ZTO nanoparticles.

Fig. 7(a–c) illustrate that the Mg_1.5_@ZTO nanoparticles exhibited the strongest inactivation efficiency, with a rate of 96.96%, compared to the others. Notably, the Mg-modified ZTO nanoparticles exhibited a similar inactivation efficiency under visible light against not only Gram-negative (*E. coli*) but also Gram-positive (*S. aureus*) bacteria strains at the same concentration and time when compared to pure ZTO, as can be seen in Fig. 7(a–c). Considering other antibacterial nanoparticles reported in the literature,^57^ the Mg-doped ZTO nanoparticles exhibited one of the highest antibacterial efficiencies for both bacteria, indicating that they might be excellent candidates for photocatalytic disinfection agents in the future.

Overall, a possible disinfection mechanism of Mg-doped ZTO nanoparticles can be mainly explained based on the aforementioned findings: primarily, the photo-induced radicals can directly attack the bacteria themselves, destroying cell membranes, causing cell matrix leakage, and ultimately leading to the death of both pathogens.^58,59^ Secondly, these radicals could possibly transform into other reactive species, such as H_2_O_2_, to further oxidize the outer membrane of bacteria.^60,61^ Third, released Mg^2+^ cationic ions show a strong affinity for primary amines and thiols groups on the surfaces of pathogens, thereby leading to the death of both bacteria.^62^

## 4. Conclusion

In this study, we successfully synthesized magnesium-doped zinc–tin oxide nanoparticles by a hydrothermal method and utilized them not only as disinfection agents to prevent the spread of Gram-positive (*S. aureus*) and Gram-negative (*E. coli*) bacteria strains but also as photocatalysts to degrade rhodamine B organic pollutant. It was clear that the ZTO nanoparticles modified with Mg^2+^ ions exhibited excellent photocatalytic and antibacterial properties under visible light, because the Mg^2+^ ions contributed significantly to a reduction in particle size, increment in surface area, suppression of nonradiative recombination, and faster separation of photogenerated charges. More importantly, the Mg cationic ions played an active role in increasing oxygen vacancies inside the crystal lattice, and particularly in the formation of superoxide radicals. The Mg_1.5_@ZTO nanoparticles exhibited extremely high inactivation efficiencies of 99.76% and 96.96%, respectively, against *E. coli* and *S. aureus* pathogens after 1 h of visible light exposure. We anticipate that future research directions will include the development of ZTO photocatalysts modified by different cationic/anionic ions and/or the utilization of magnesium as an additive into various oxide-based compositions and/or application in various experiments for gaining an in-depth understanding of the mechanisms underlying bacterial disinfection to promote their more considerable efficiency and ensure high stability for future large-scale commercialization.

## Data availability

Data supporting this study are included within the article and/or ESI.†

## Conflicts of interest

The authors declare that they have no known competing financial interests or personal relationships that could have appeared to influence the work reported in this paper.

## Supplementary Material

NA-OLF-D4NA00811A-s001
