# Supplementary material for: Ternary zinc–tin-oxide nanoparticles modified by magnesium ions as a visible-light-active photocatalyst with highly strong antibacterial activity
Source: Nanoscale Adv. 2024 Nov 11;6(23):6008–18. doi: 10.1039/d4na00811a (PMC11575554; doi:10.1039/d4na00811a)
Supplement: NA-006-D4NA00811A-s001 [file NA-006-D4NA00811A-s001.pdf]

Supplementary Information (SI) for Nanoscale Advances.
This journal is © The Royal Society of Chemistry 2024

Supporting Information

Ternary Zinc-Tin-Oxide nanoparticles modified by magnesium ions as a visible-light-active photocatalyst for highly strong antibacterial activity

Alaa KAMO[a,b], Ozlem ATES SONMEZOGLU [b] and Savas SONMEZOGLU[a,c*]

[a] Nanotechnology R&D Laboratory, Karamanoglu Mehmetbey University, 70100, Karaman, Türkiye

[b] Department of Bioengineering, Karamanoglu Mehmetbey University, 70100, Karaman, Türkiye

[c] Department of Metallurgical and Materials Engineering, Karamanoglu Mehmetbey University, 70100, Karaman, Türkiye

*Corresponding Author: svssonmezoglu@kmu.edu.tr

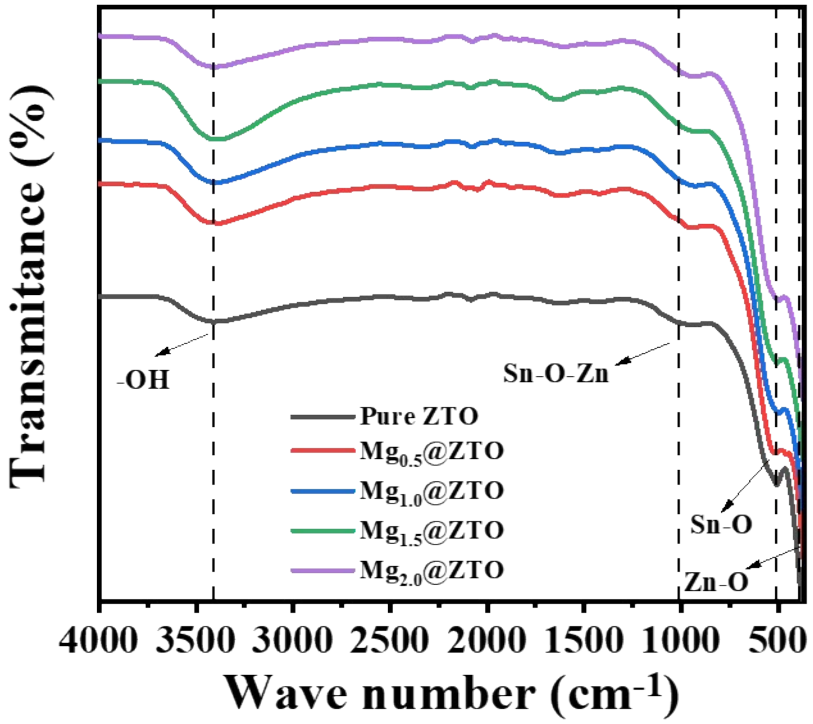

S-1: Fourier transform infrared (FTIR) spectra of pure and Mg-doped ZTO nanoparticles.

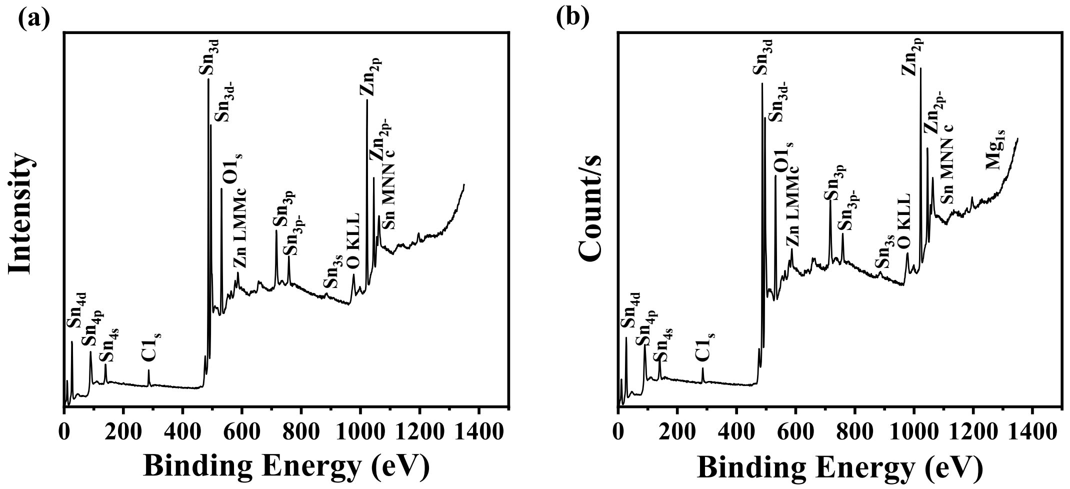

S-2: X-ray photoelectron spectroscopy (XPS) analysis, full scan spectrum of pure and $Mg_{2.0}$@ZTO nanoparticles.

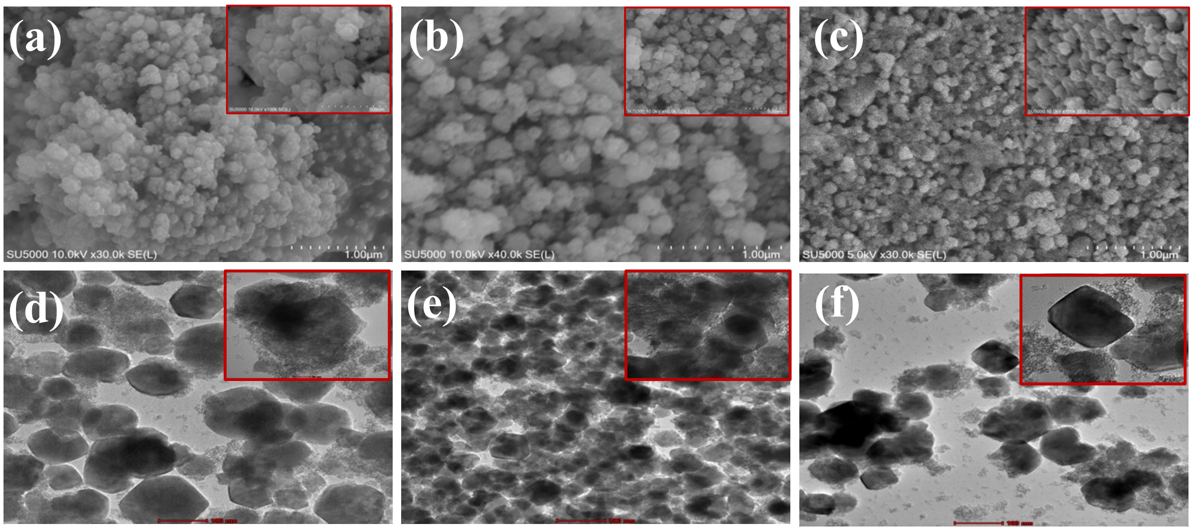

S-3: SEM images of (a) $Mg_{0.5}$@ZTO, (b) $Mg_{1.0}$@ZTO and, (c) $Mg_{2.0}$@ZTO nanoparticles, TEM images of (d) $Mg_{0.5}$@ZTO, (e) $Mg_{1.0}$@ZTO and, (f) $Mg_{2.0}$@ZTO nanoparticles.

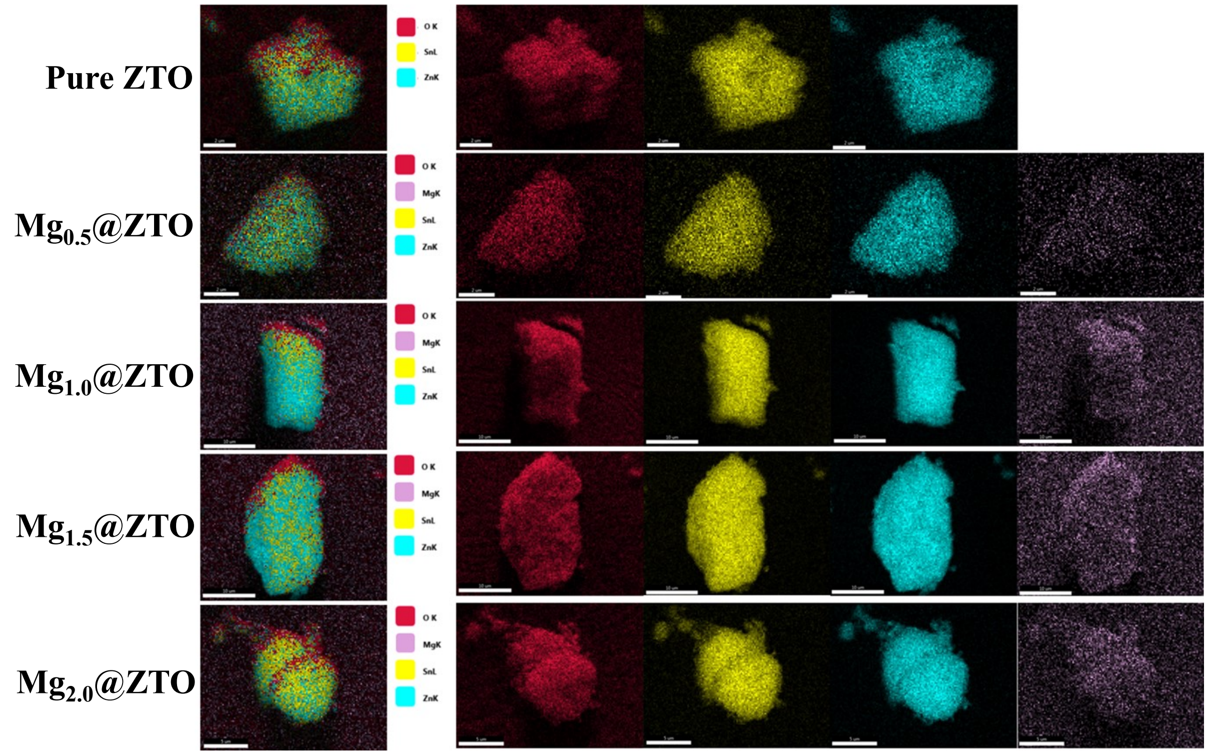

S-4: Elemental mapping of Zn, Sn, and O of pure and Mg-doped ZTO nanoparticles.

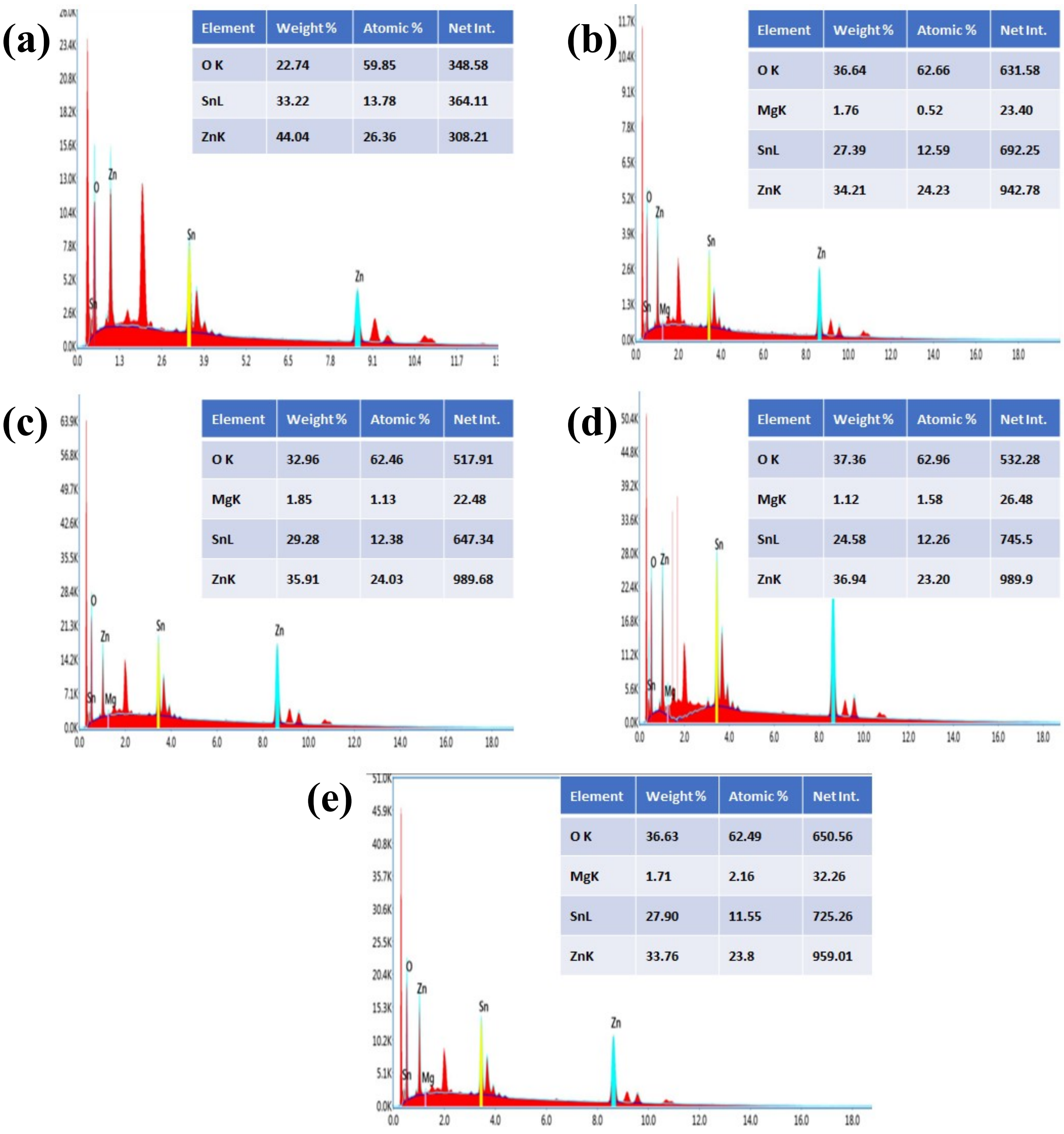

(a)

Element	Weight %	Atomic %	Net Int.
O K	22.74	59.85	348.58
SnL	33.22	13.78	364.11
ZnK	44.04	26.36	308.21

(b)

Element	Weight %	Atomic %	Net Int.
O K	36.64	62.66	631.58
MgK	1.76	0.52	23.40
SnL	27.39	12.59	692.25
ZnK	34.21	24.23	942.78

(c)

Element	Weight %	Atomic %	Net Int.
O K	32.96	62.46	517.91
MgK	1.85	1.13	22.48
SnL	29.28	12.38	647.34
ZnK	35.91	24.03	989.68

(d)

Element	Weight %	Atomic %	Net Int.
O K	37.36	62.96	532.28
MgK	1.12	1.58	26.48
SnL	24.58	12.26	745.5
ZnK	36.94	23.20	989.9

(e)

Element	Weight %	Atomic %	Net Int.
O K	36.63	62.49	650.56
MgK	1.71	2.16	32.26
SnL	27.90	11.55	725.26
ZnK	33.76	23.8	959.01

S-5: EDX spectrum of (a) pure ZTO, (b) $Mg_{0.5}$@ZTO, (c) $Mg_{1.0}$@ZTO, (d) $Mg_{1.5}$@ZTO, and (e) $Mg_{2.0}$@ZTO nanoparticles.

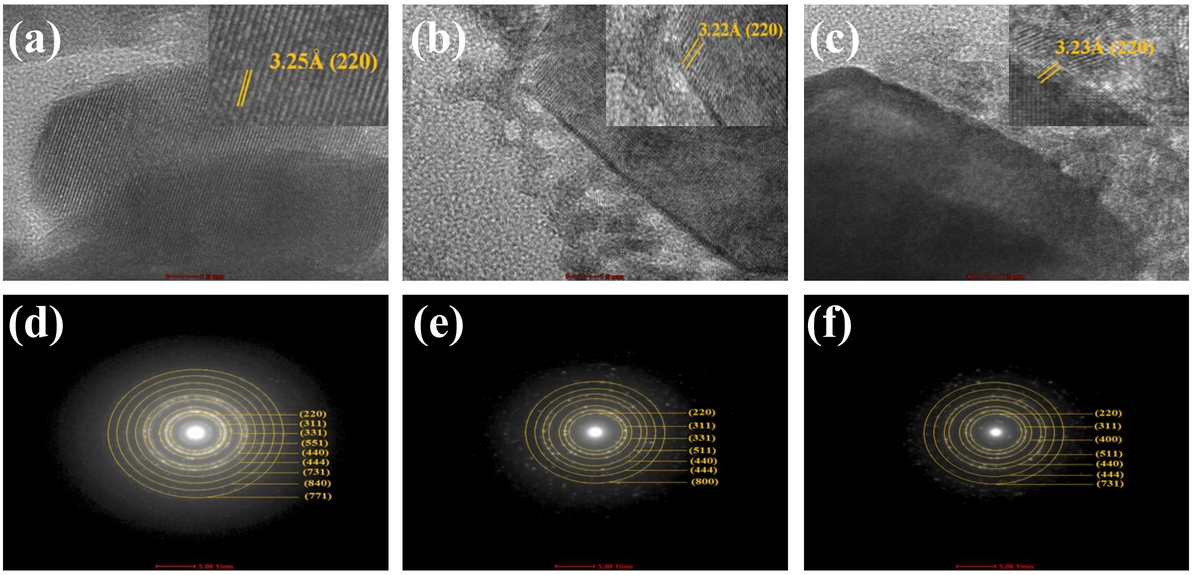

S-6: HRTEM and SAED analyis of (a,d) $Mg_{0.5}$@ZTO, (b,e) $Mg_{1.0}$@ZTO and, (c,f) $Mg_{2.0}$@ZTO nanoparticles.

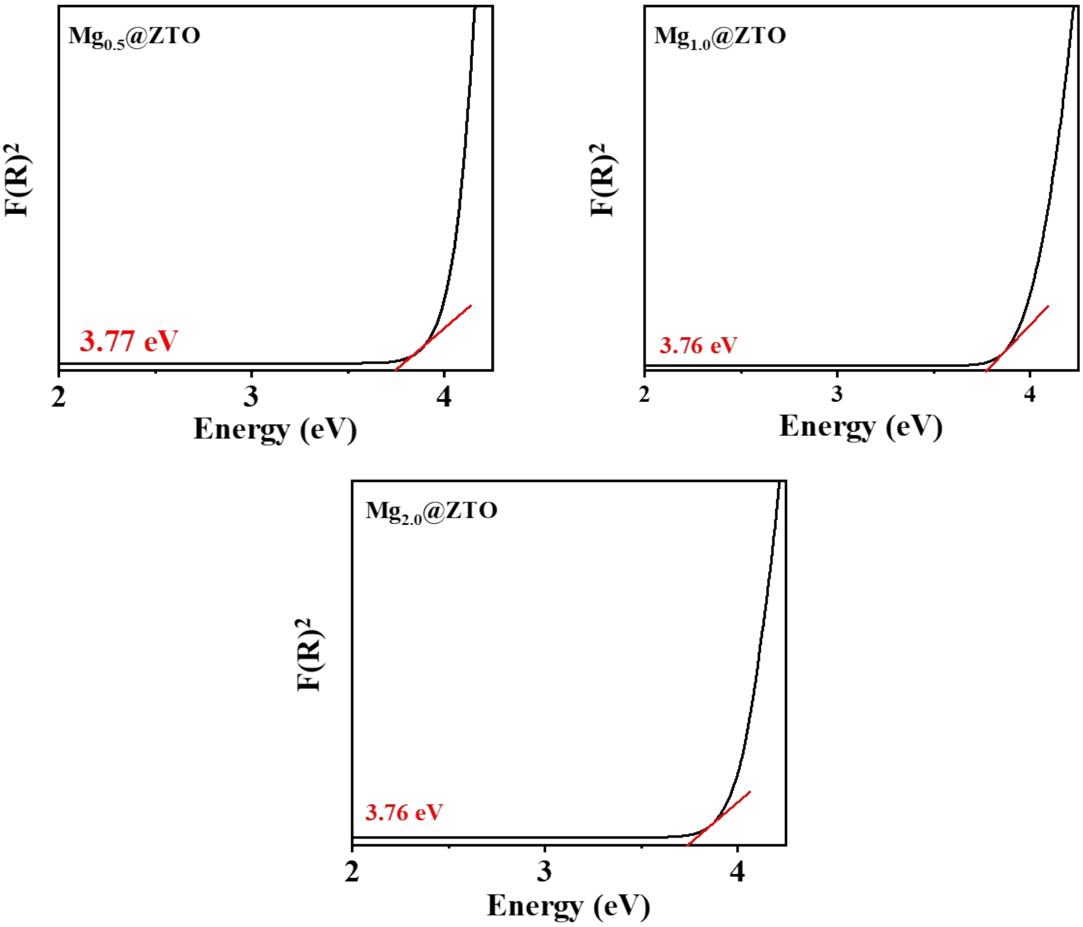

S-7: Band gap of (a) $Mg_{0.5}$@ZTO, (b) $Mg_{1.0}$@ZTO and, (c) $Mg_{2.0}$@ZTO nanoparticles.

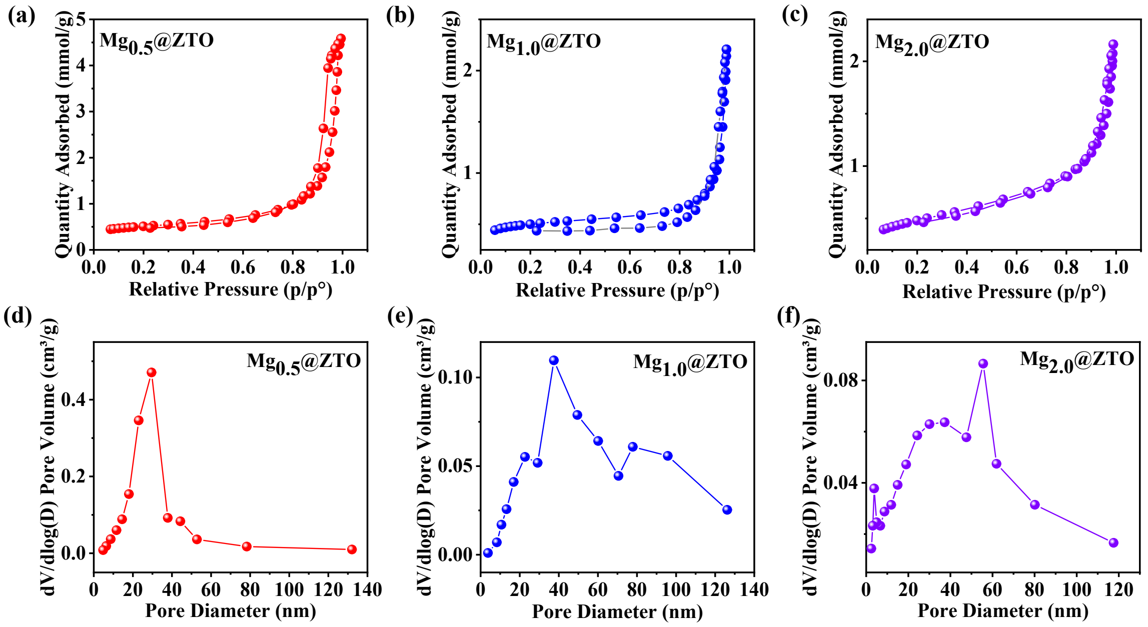

S-8: N_2 adsorption-desorption isotherm curve and pore diameter of (a,d) $Mg_{0.5}$@ZTO, (b,e) $Mg_{1.0}$@ZTO and, (c,f) $Mg_{2.0}$@ZTO nanoparticles.

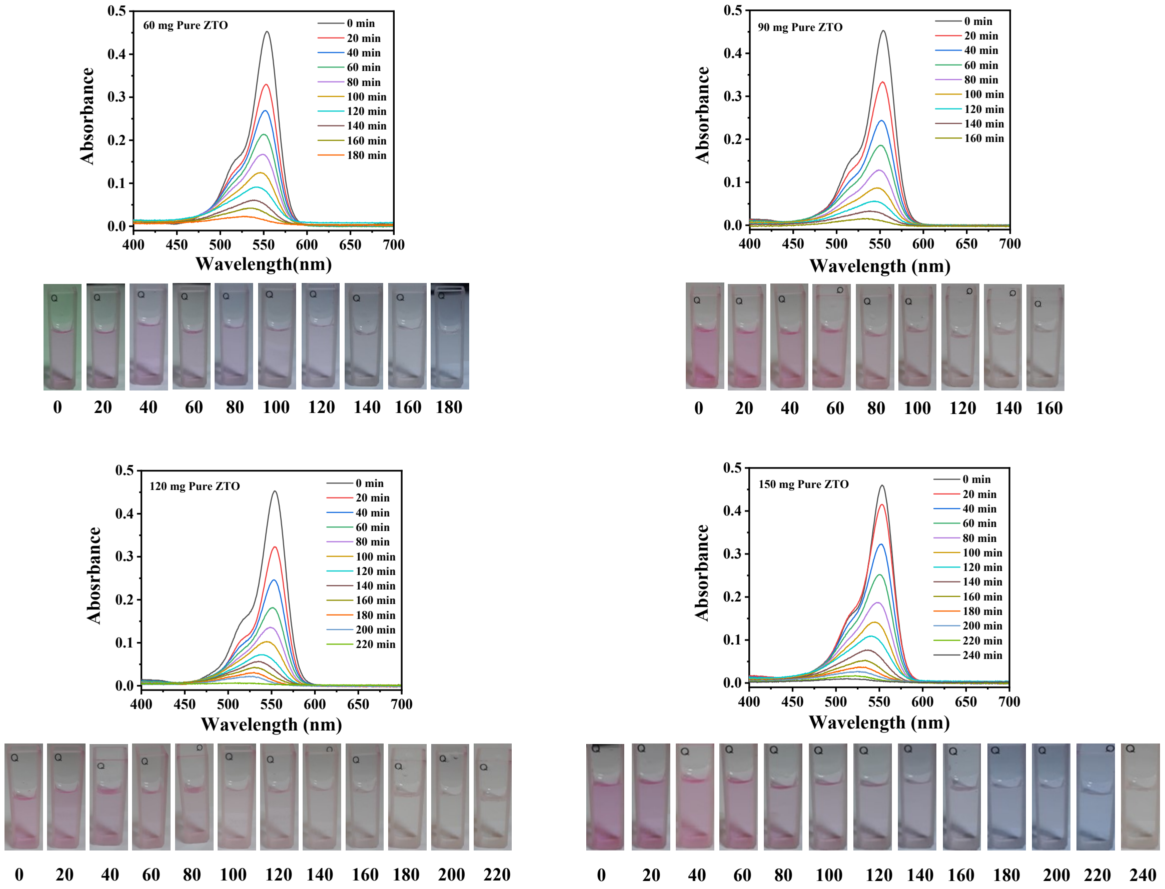

S-9: Real-time UV–vis absorption spectra of the degradation of RhB solutions containing different amount of pure ZTO nanoparticles.

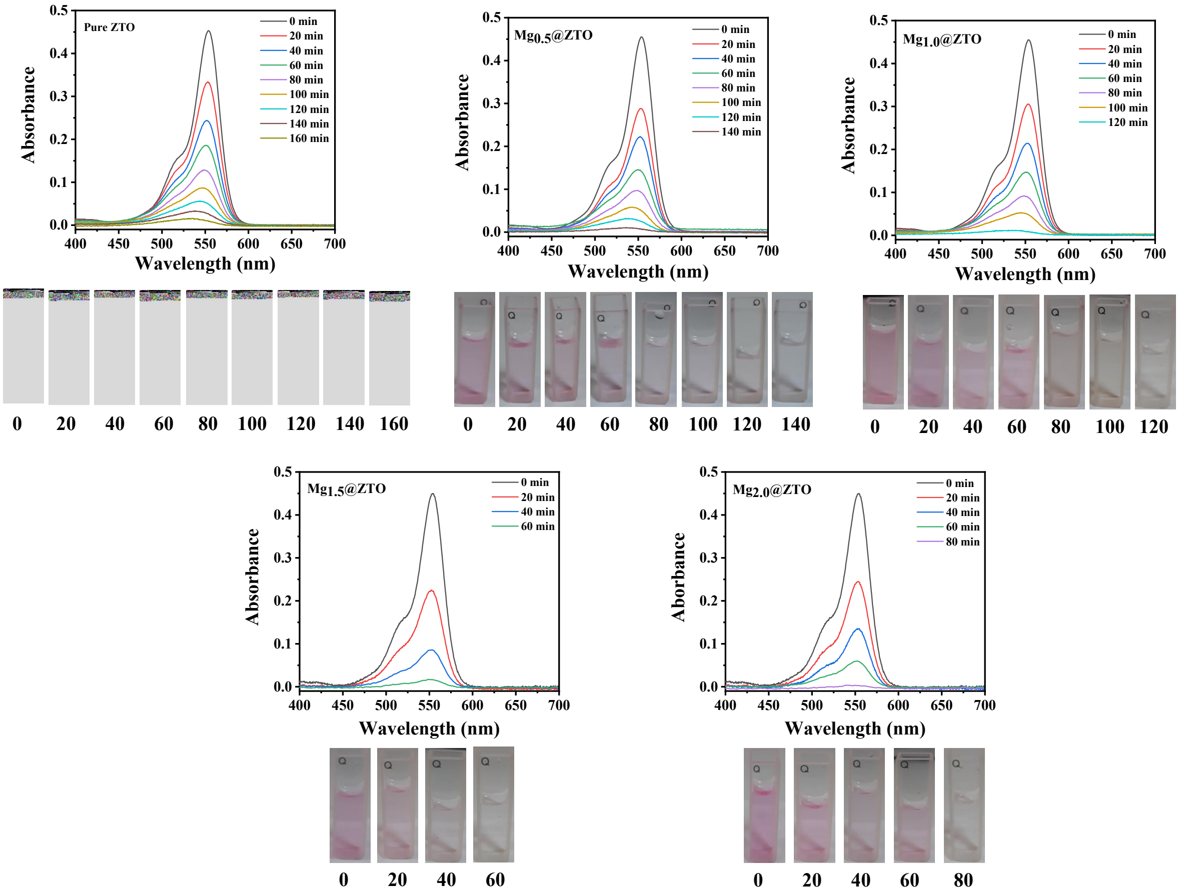

S-10: Real-time UV–vis absorption spectra of the degradation of RhB solutions in the present the pure and Mg-doped ZTO nanoparticles under UV light illumination.

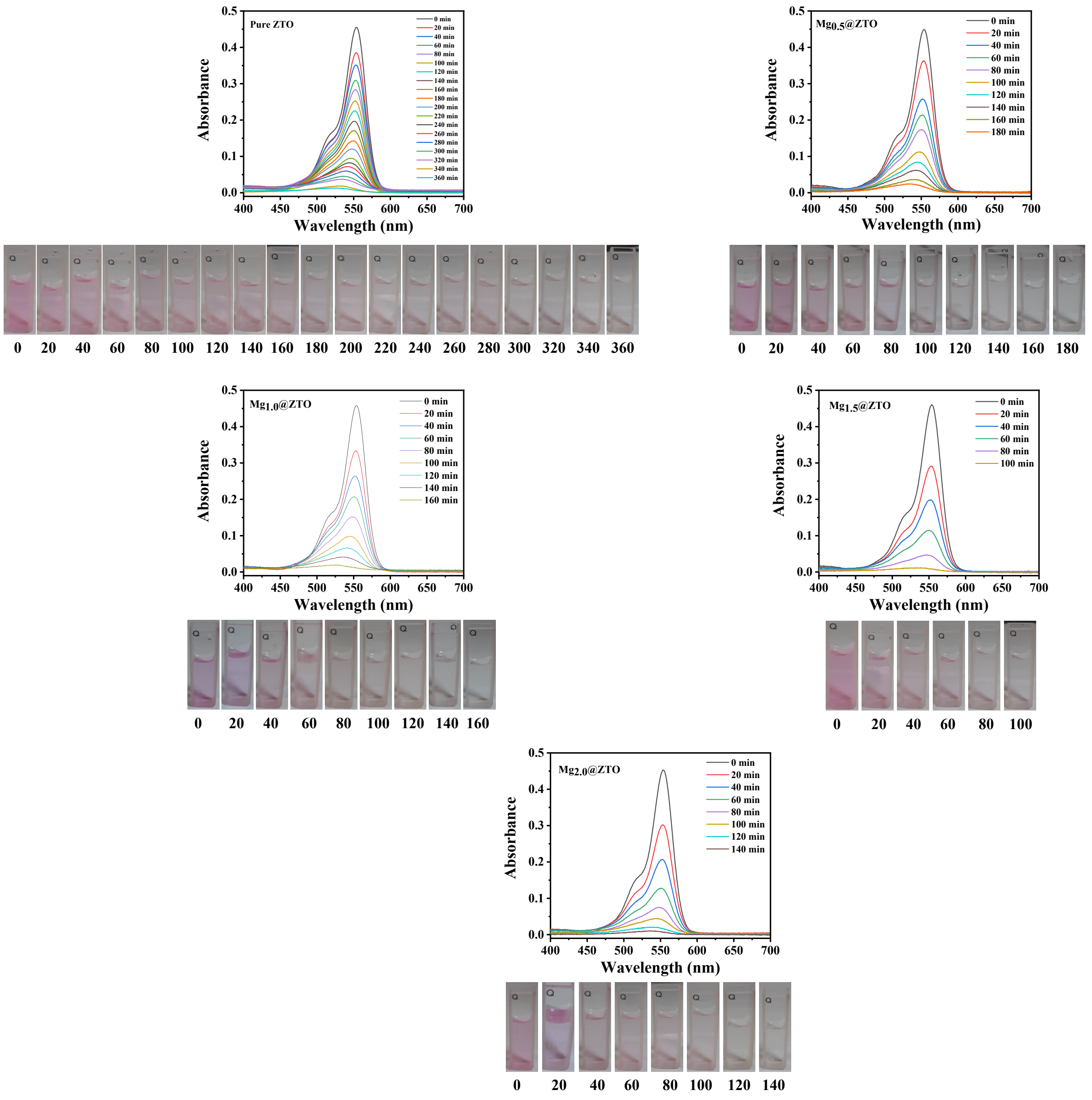

S-11: Real-time UV–vis absorption spectra of the degradation of RhB solutions in the present of the pure and Mg-doped ZTO nanoparticles under visible light illumination.

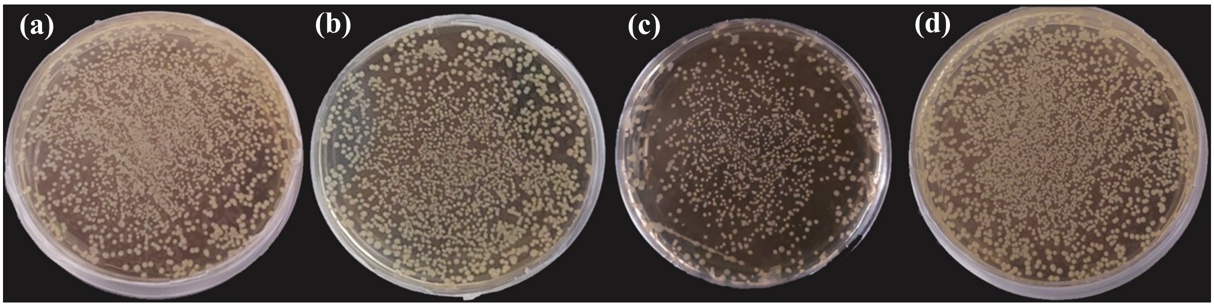

S-12: Effect of amount of pure ZTO nanoparticles on antibacterial activity against *E. Coli* a) control, b) 500 μL, c) 1000 μL and d) 2000 μL.

Table S1: A brief summary of the photocatalytic and antibacterial performance for various dopants based on Zn_2SnO_4 in the literature.

Nanoparticle	Light Source	Catalyst Amount/ Concentration	Organic Pollutant	Degradation Rate/Time	Antibacterial activity	Reference
Co-ZTO	Visible light	1 mg/mL	RhB	93%/120 min	-	1
Co-ZTO	Ultraviolet light	0.1 M	MB	~80%/ 6 hours	-	2
S-ZTO	Visible light	-	RhB	92%/40 min	-	3
Yb-ZTO	Visible light	1mh/mL	MB	97.1%/ 120 min	-	4
Nd-ZTO	Xenon light	0.4 g/L	AR14	21.9%/90 min	-	5
Er-ZTO	Xenon light	0.4 g/L	AR14	87.4%/90 min	-	5
Gr-ZTO	Xenon light	0.4 g/L	AR14	72.2%/90 min	-	5
Ag-ZTO	Ultraviolet light	0.1 mg/mL	MB	83.1%/ 60 min	-	6
Ag-ZTO	Visible light	-	MB	96%/120 min	-	7
Mn-ZTO	Visible light	0.25 g/L	RhB	~ 80%/ 120 min	-	8
Gd-ZTO	Visible light	0.25 g/L	RhB	~60%/120 min		8
Co-Fe-ZTO	-	2 mg/mL	-	-	*S. aureus* (22 mm) *E. coli* (30 mm)	9
Ca-ZTO	-	2 mg/mL	-	-	*K. pneumoniae* (99%)	10
Mg-ZTO	Visible light	0.9 mg/mL	RhB	98%/ 100 min		**This Work**
		1 mg/mL			*S. aureus* (96.96%) *E. coli* (99.76%)	

References:

1. X. Hu, H. Hao, W. Guo, S. Jin, H. Li, H. Hou, G. Zhang, S. Yan, W. Gao and G. Liu, *Chemical Physics*, 2017, **490**, 38-46.
2. I. Y.-Y. Bu, *Ceramics International*, 2014, **40**, 8103-8109.
3. Y. Lin, S. Lin, M. Luo and J. Liu, *Materials Letters*, 2009, **63**, 1169-1171.
4. J. Xue, D. Lei, Q. Bi, C. Tang and L. Zhang, *Optical Materials*, 2020, **108**, 110454.
5. A. R. Amani-Ghadim, S. Arefi-Oskoui, A. Karimi, A. Khataee, F. F. Azhar, A. T. Sareshkeh and M. S. S. Dorraji, *Journal of Alloys and Compounds*, 2023, **934**, 167837.
6. Q. Lu, Z. Wei, C. Li, J. Ma and L. Li, *Materials Science in Semiconductor Processing*, 2022, **138**, 106290.
7. J.-C. Su, T.-L. Hsieh, S.-M. Yang, S.-C. Chao and K.-C. Lu, *Nanomaterials*, 2022, **12**, 1201.
8. M. B. Ali, R. Nasser, S. M. Alshahrani, H. A. Al-Shamiri, B. Elgammal and H. Elhouichet, *Ceramics International*, 2021, **47**, 32882-32890.
9. R. K. Hameed, S. M. AL-Jawad and N. J. Imran, *Optical and Quantum Electronics*, 2024, **56**, 1122.
10. A. Pandimurugan, G. V. Prasath, K. Usha, J. Vivekanandan, C. Karthikeyan, K. Sankaranarayanan and G. Ravi, *Applied Physics A*, 2023, **129**, 154.
